# Post-stroke cognitive impairment and brain hemorrhage are augmented in hypertensive mice

**DOI:** 10.1177/0271678X241262127

**Published:** 2024-06-17

**Authors:** David E Wong Zhang, Tayla A Gibson Hughes, Hericka B Figueiredo Galvao, Cecilia Lo, Quynh Nhu Dinh, Shenpeng R Zhang, Hyun Ah Kim, Sharmalee Selvaraji, Andrew N Clarkson, Thiruma V Arumugam, Grant Drummond, Christopher G Sobey, T Michael De Silva

**Affiliations:** 1 Centre for Cardiovascular Biology and Disease Research and La Trobe Institute for Molecular Sciences (LIMS), La Trobe University, Victoria, Australia; 2 Department of Microbiology, Anatomy, Physiology & Pharmacology, School of Agriculture, Biomedicine, Environment, La Trobe University, Victoria, Australia; 3 Department of Physiology, Yong Loo Lin School Medicine, National University of Singapore, Singapore, Singapore; 4 Memory Aging and Cognition Centre, Department of Pharmacology, Yong Loo Lin School of Medicine, National University of Singapore, Singapore, Singapore; 5 NUS Graduate School for Integrative Sciences and Engineering, National University of Singapore, Singapore, Singapore

**Keywords:** Cognitive impairment, hypertension, ischemic stroke, neuroinflammation, vascular dementia

## Abstract

Hypertension is a major risk factor for both stroke and cognitive impairment, but it is unclear whether it may specifically affect post-stroke cognitive impairment. We assessed the effect of hypertension and/or stroke on brain injury, cognitive outcome, and the brain transcriptomic profile. C57BL/6J mice (n = 117; 3–5 mo.) received s.c. infusion of either saline or angiotensin II followed by sham surgery or photothrombotic stroke targeting the prefrontal cortex seven days later. Cognitive function was assessed with the Barnes maze and RNA sequencing was used to quantify transcriptomic changes in the brain. Angiotensin II treatment produced spontaneous hemorrhaging after stroke. In the Barnes maze, hypertensive mice that received stroke surgery had an increased escape latency compared to other groups (day 3: hypertensive + stroke = 166.6 ± 6.0 s vs. hypertensive + sham = 122.8 ± 13.8 s vs. normotensive + stroke = 139.9 ± 10.1 s vs. normotensive + sham = 101.9 ± 16.7 s), consistent with impaired cognition. RNA sequencing revealed >1500 differentially expressed genes related to neuroinflammation in hypertensive + stroke vs. normotensive + stroke, which included genes associated with apoptosis, microRNAs, autophagy, anti-cognitive biomarkers and Wnt signaling. Overall, we show that the combination of hypertension and stroke resulted in greater learning impairment and brain injury.

## Introduction

Dementia describes the deterioration of one or more neurological symptoms associated with cognitive impairment— characterized as deficits in language, memory, perceptual or motor praxis, social cognition, executive or visuospatial functioning.^1^ It is a leading cause of death and disability worldwide, and the burden of dementia is expected to almost triple in the coming decades.^2^ Current pharmacological treatments to improve cognition in individuals with dementia are limited,^3^ they primarily treat symptoms and are thus not disease-modifying.^4^ Another major limitation of current treatment options is that pharmacotherapies are being developed for Alzheimer’s pathology and not suited for dementias-linked to other neurological conditions. It should be noted that different biomarker strategies and interventions need to be developed for the detection and treatment of different types or cognitive impairment.^5^ Thus, the development of new therapies is clearly needed to address the incidence of this progressive neurodegenerative disease. A major roadblock to the development of new therapies is our current lack of understanding of dementia and its underlying mechanisms.^6^

While Alzheimer’s pathology is a major contributing factor, many cases of dementia are thought to be a consequence of vascular-related diseases. Thus, many patients present with ‘mixed dementia’.^7^ Highly prevalent cardiovascular disorders such as hypertension and stroke are not only major causes of premature mortality and morbidity^8,9^ but are known contributors to brain injury and development of cognitive decline.^10,11^ Hypertension has numerous adverse effects on the brain, including neuroinflammation and dysregulation of cerebral blood flow which increases the risk of stroke.^12^ In addition to ischemia, stroke itself may result in cerebrovascular dysfunction, white matter lesions, and neurodegeneration— all of which may further increase the risk of post-stroke cognitive impairment and dementia.^10^ Dementia, hypertension, and stroke are therefore closely connected,^13^ but few studies have sought to test for combined effects of hypertension and stroke on cognition.

In this study, we utilized the angiotensin II model of hypertension in conjunction with the photothrombotic model of prefrontal cortical stroke to test for effects of these common comorbidities on brain health. We hypothesized that hypertension would exacerbate post-stroke cognitive impairment and brain injury.

## Methods

### Experimental animals

**Figure 1.**
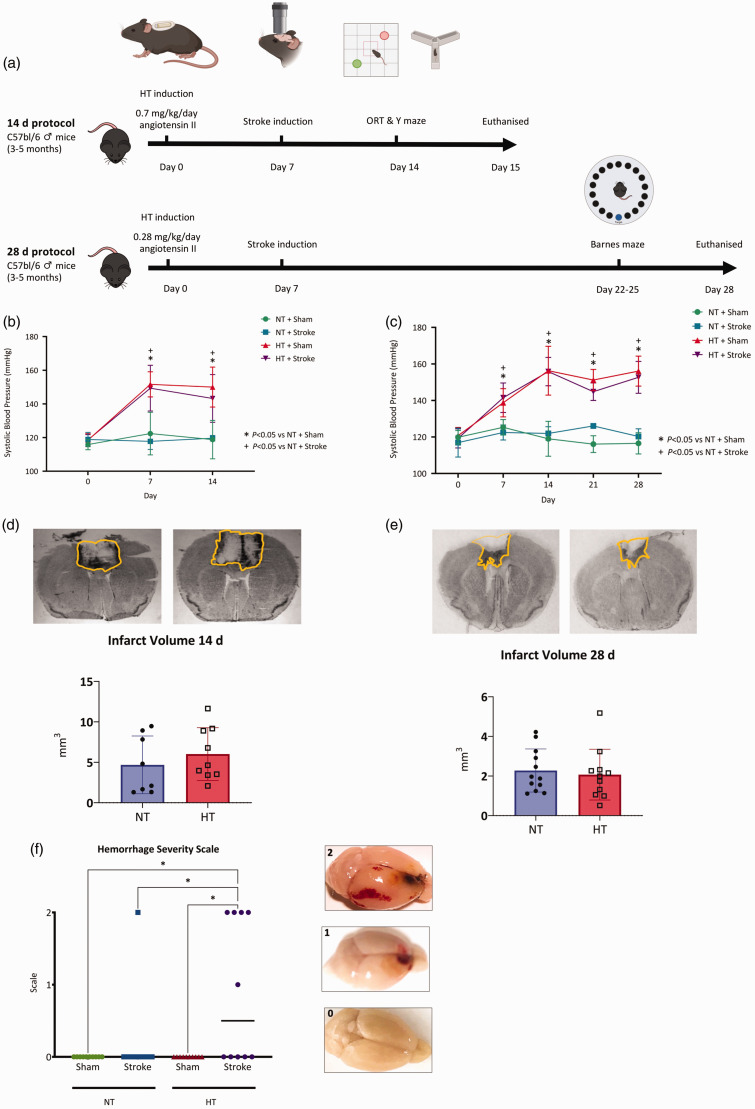
Systolic blood pressure, infarct volume and occurrence of haemorrhage. (a) Experimental timeline for 14- and 28-day protocols performed in this study. (b) Systolic blood pressure (mmHg) measured on day 0, 7 and 14 via tail-cuff plethysmography. Continued.Normotensive mice (NT) were infused with 0.9% saline whilst hypertensive mice (HT) were infused with angiotensin II at 0.7 mg/kg/day. (c) Systolic blood pressure (mmHg) measured on day 0, 7, 14, 21 and 28 via tail-cuff plethysmography. Normotensive mice (NT) were infused with 0.9% saline whilst the hypertensive mice (HT) were infused with angiotensin II at 0.28 mg/kg/day. Representative images (top) and quantification of infarct volume (mm^3^) in NT + Stroke and HT + Stroke mice at (c) 14 or (d) 28 days. (f) Occurrence of hemorrhages across all groups (left) with representative images of hemorrhage severity (right). (b and c) Data are presented as mean ± SD. N = 9–13/group. * = *P < *0.05 HT + Sham vs NT + Sham and + indicates *P < *0.05 HT stroke vs NT + Stroke, two-way ANOVA mixed-effects analysis with Geisser-Greenhouse correction with multiple comparisons. (d and e) Data are presented as mean ± SD, n = 8–12/group, unpaired t-test. (f) Median value is indicated, n = 10–12/group. * Indicates *P < *0.05, Kruskal-Wallis test.

All procedures were approved by the La Trobe University Animal Ethics Committee (AEC16-79). All procedures were performed in accordance with the ARRIVE guidelines and the National Health and Medical Research Council of Australia code for the care and use of animals for scientific purposes. Male C57BL/6J mice (3–5 months old) were purchased from Animal Resource Centre (Canning Vale, Western Australia) and housed in a controlled 12-hour light/dark cycle (7:00 am to 7:00 pm) with access to food and water *ad libitum*. Summary of the experimental timeline for both the 14-day and 28-day protocol are shown in Figure 1(a).

### Infusion of vehicle or angiotensin II and measurement of blood pressure

We used the angiotensin II infusion model of hypertension which is one of the most widely used and characterized methods for the study of hypertension in mice. This model simulates a hyperactive renin-angiotensin aldosterone system and has been shown to promote inflammation and altered cerebrovascular structure and function.^14^ Mice were randomly assigned to receive subcutaneous infusion of either 0.9% saline (normotensive, NT) or angiotensin II (hypertensive, HT). HT mice were infused with angiotensin II at 0.7 mg/kg/day for 14 days via a two-week ALZET osmotic pump (ALZET-2002 pumps; DURECT Corporation, USA) implanted subcutaneously in the right flank whilst under anesthesia.^15^ We also examined the effects of hypertension at 0.28 mg/kg/day for 28 days (ALZET-2004 pumps). Angiotensin II has been shown to induce cognitive impairment in rodent models of hypertension.^16^ However, it is unknown if the pressor dose of angiotensin II (0.7 mg/kg/day) would mask the potential synergistic effect of hypertension and stroke on cognition. Thus, a lower dose (0.28 mg/kg/day), previously shown to elevate systolic pressure after approximately 3 weeks in young male mice,^15^ was administered for 4 weeks. Systolic blood pressure was measured between 7:00 am to 12:00 pm by tail cuff plethysmography as previously described.^17^

### Laser speckle contrast imaging

Cerebral blood flow (CBF) in the cortex was measured using laser speckle contrast imaging (LSCI; Pericam PSI HR 90-00044, Perimed AB, Sweden) at baseline and prior to euthanasia in mice undergoing the 14-day protocol. Mice were anaesthetized with isoflurane (5% induction, 1.5% maintenance in 100% O_2_) the head was shaved and a 2 cm incision was made in the skin overlying the cranial midline. This dose of isoflurane has been shown to support stable, near-physiological cardiovascular function while providing acceptable depth of anaesthesia.^18^ Average cerebral blood flow over 60 s (1 image taken every 2 s) in a 20 mm^2^ region of interest between the frontonasal suture and bregma, which included the prefrontal cortex, was calculated using PIMsoft software Version 1.5 (Perimed AB, Sweden). CBF was measured at baseline (before implantation of osmotic pumps) and prior to euthanasia. The relative perfusion deficit was also quantified 14 days after stroke.

### Photothrombotic stroke

In this study, we used the photothrombotic model of ischemic stroke. This is a commonly used model that allows the targeting of stroke to a specific region of the cortex, produces reproducible infarct size, has a low mortality rate and thus enables the study of long-term functional outcomes. Photothrombosis was used to induce ischemic stroke at one-week post-osmotic pump implantation.^19^ All mice were confirmed as hypertensive prior to photothrombotic stroke (Figure 1(b) and (c)). Mice were anaesthetized, fur removed, the skin was sterilized at the surgical site, a midline incision was made on the scalp and bregma was identified. Mice were then placed in a stereotaxic frame (David Kopf Instruments, USA). A cold light source (KL 1600 LED, Schott) attached to a 40x objective lens was positioned centrally at 1.2 mm anterior to bregma to target the prefrontal cortex. Mice were then injected i.p. with 2 mg of Rose Bengal in 0.2 mL (10 mg/mL in 0.9% saline) which was allowed to circulate for 5 min. The prefrontal cortex was then illuminated for 18 min at 680 lumens over 2 mm^2^ area. Mice receiving sham surgery underwent the same procedures except for illumination.^19^

Thus, there were 4 treatment groups: normotensive mice that received sham surgery (NT + Sham; n = 22); normotensive mice that received stroke surgery (NT + Stroke; n = 19); hypertensive mice that received sham surgery (HT + Sham, n = 23); and hypertensive mice that received stroke surgery (HT + Stroke; n = 25). Group numbers are shown in Supplementary Table 1.

### Behavioral tests

Mice assigned to the 14-day protocol underwent two consecutive behavioral tests, the object location task (days 12–14) followed by the spontaneous alternation task (day 14; 1 hour after the object relocation task). Mice assigned to the 28-day protocol had their cognition assessed via the Barnes maze (days 22–25). A summary of the experimental timeline is shown in Figure 1(a).

#### Object relocation task

The object relocation task was performed in an opaque 40 × 40 cm box and consisted of 3 phases: habituation, training, and retention. During the habituation phase, mice were placed in the empty box for 10 min per day for two days prior to training. During the training phase, mice were placed in the box which contained two identical objects for 10 min before returning them to their home cage. One hour later, mice were returned to the box with one of the objects relocated to a new position and given 5 min to explore the objects. The maze and objects are cleaned with 70% ethanol between trials to remove olfactory stimuli. Ethovision XT software (Noldus Instruments, Version 10) was used for automated quantification of exploratory behavior. A 2 cm interaction zone around each object was set up and exploration of an object was defined as the mouse’s nose entering these zones. Animals that did not explore objects for at least 3 sec for both objects in either the training or retention phase were excluded from analyses.^20^ The amount of time exploring the original vs relocated object was calculated as a percentage of the total exploration time for both objects.

#### Spontaneous alternation test

The spontaneous alternation test comprises of three arms, each at 120° from one another (arms A, B and C). Distinct cues are placed at the end of each arm to allow for recognition of an area previously explored. A mouse is allowed 5 min to freely explore. Alternation was defined as the consecutive entries into arms of the Y maze not previously explored (i.e. entry into arm A then B then C = 1 alternation). The maze was cleaned with 70% ethanol between trials to remove olfactory stimuli. Video footage was manually analyzed.

#### Barnes maze

The Barnes maze consists of a circular platform (1 m diameter) with 20 identical circles surrounding the perimeter. Visual cues were placed around the maze to assist mice with orientation. Indirect bright lights and a continuous buzzer set to 1 kHz at 85–90 dB are used as aversive stimuli. Each mouse was randomly assigned to an escape hole located in the same position for each trial and day. Mice were kept in a dark room adjacent to the testing room for 20 min intervals between trials, with 3 trials performed per mouse for three consecutive days. During the acquisition phase, mice were allowed 180 s to locate and enter the escape hole. If the escape hole had not been entered within the 180 s, the mouse was guided to the escape box. Once inside, the escape hole was covered for 60 s and the buzzer was switched off. The mouse was returned to its cage until the next trial. The platform and escape box were cleaned with 70% ethanol between trials to remove olfactory stimuli. To assess spatial memory, learning and locomotive activity, the following parameters were measured: (1) primary latency (time taken to reach the escape hole), (2) escape latency (time taken to enter the escape hole), (3) pathlength (distance travelled) (4) primary errors (error visits before locating the escape hole) and (5) velocity, all metrics were averaged across the three trials for each day. All parameters were automatically tracked using Ethovision XT (Noldus Instruments, Version 15.0).

## Post-mortem analyses

All mice were perfused with 0.01 M phosphate-buffered saline (PBS) containing 0.2% clexane (400 IU, Sanofi Aventis, Australia) via the left ventricle (15 mL/min) to remove intravascular blood. Mice were euthanized between 8 AM and 12 noon to minimize circadian effects.

### Hemorrhage grading

Brains were collected and hemorrhage severity was graded based using a 3-point scoring system. A score of 0 indicated no evidence of hemorrhage, a score of 1 indicated hemorrhage within the infarct region, and a score of 2 indicated hemorrhage outside of the infarct region.

### Calculation of infarct volume

Thirty µm coronal sections, 210 µm apart, were cut using a Leica CM1850 cryostat. Sections were stained with thionin^21^ and imaged using TCapture software (Version 5.1, Tucsen Photonics) connected to a Leica WILD M3Z microscope. Infarct volume was quantified using ImageJ software (NIH) via the following equation: *Infarct volume = infarct area × section thickness*. Infarct volumes were summed across all sections to determine total infarct volume.

## Immunofluorescence staining and imaging of IgG and GFAP

Ten μm coronal sections of the prefrontal cortex which contained the infarct were fixed in 100% acetone and washed in 0.01 M PBS for 5 min. Sections were blocked with 10% goat serum containing 0.2% triton-X or 1 hour and then incubated with rabbit anti- glial fibrillary acidic protein (GFAP) antibody (ab116010, Abcam; 1:500) overnight at 4 °C. Sections were then incubated in goat anti-rabbit secondary antibody (Alexa 594, 1:200, Thermo Fisher Scientific), washed, mounted with Vectashield mounting media (Vector Laboratories), coverslipped and stored at 4 °C. Sections of the pre-frontal cortex and hippocampus were also stained for deposition of IgG (marker of blood-brain barrier injury). For IgG, sections were fixed as described above and incubated with a goat anti-mouse secondary antibody (Alexa 555, 1:200, Thermo Fisher Scientific) washed, mounted with Vectashield mounting media Vector Laboratories), coverslipped and stored at 4 °C. Images were acquired at 400X using an Olympus BX53 microscope running CellSens software (version 1.18).

GFAP-stained images were captured at bilateral regions of the infarct. Images were acquired at 400X using an Olympus BX53 microscope running CellSens software (version 1.18). Images for sham mice were taken from equivalent locations. For GFAP analysis, we used the skeletonization analysis of GFAP as a marker of reactive astrocytes. Images were converted into 8-bit binary and thresholded for the segmentation of pixels. A skeleton analysis script on FIJI was used to convert images into a skeletal figure. Each skeleton represents an astrocyte with its processes. The total number of branches and branch lengths were averaged across all images to estimate astrocyte activation. IgG-stained images were captured surrounding the infarct in the prefrontal cortex. Location was kept consistent between mice. Each image was thresholded then averaged across all images to determine integrated density using FIJI.

## RNA extraction and validation

RNA was extracted from a coronal section of brain that included the prefrontal cortex. RNA was extracted from the brains of mice undergoing the 28-day protocol. Frozen brain tissue spanning the prefrontal region was isolated and homogenized in TRIzol (Life Technologies). To synthesize cDNA, total RNA was extracted via Qiagen RNeasy mini kit as previously described.^15^ RNA concentration and quality was assessed using a NanoDrop spectrophotometer (Thermo Fisher Scientific). Only samples with a 260/280 ration of ≥1.8 was used for subsequent analysis.

## RT-PCR gene analysis

First-strand cDNA was converted from RNA using a high-capacity cDNA RT Kit (Applied Biosystems, USA). Commercially available primers (Applied Biosystems, USA; Supplementary Table 2) were used to measure gene expression of and a house-keeping gene, GAPDH, on a CFX96 Touch Real-Time PCR Detection machine (Bio-Rad, USA). Changes in gene expression were assessed using the delta-delta C_T_ method.^22^

## Differentially expressed gene analysis

RNA sequencing was performed as previously described.^23^ RNA was sent to NovogeneAIT Genomics (Singapore) for cDNA library preparation and RNA sequencing. mRNA was purified from total RNA using poly-T oligo-attached magnetics. mRNA was converted to cDNA and purified using AMPure XP Beads (Beckman Coulter Life Sciences, USA). cDNA libraries were acquired by PCR amplification. High-throughput sequencing was conducted using the HiSeqTM2500 platform (Illumina, USA). The results were mapped to the Ensembl-released mouse genome sequence and annotation. Differential expression analysis was conducted using the DESeq R Package V.1.10.1 and P-values were adjusted using the Banjamini and Hochberg’s approach for controlling the false discovery rate. Genes were considered differentially expressed if the adjusted P-value was less than 0.05. For heat map and GObar analyses, differentially expressed genes (DEGs) were filtered through databases related to immunity and inflammation (Innate Immunity Genes, Immport, Immunome database, MAPK/NFKB Network, Septic Shock Group). R studio (version 4.2.2) packages: gplots, seurat, and biocmanager were used to generate enhanced heat maps. Gene ontology (GO) enrichment analyses were performed using the org.Mm.eg.db package (bioconductor), p-adj threshold was set to 0.01. Entrez Gene identifiers were used to map DEG count with corresponding GO terms relating to biological processes. Venn diagrams were generated by a bioinformatics and evolutionary genomics tool (https://bioinformatics.psb.ugent.be/webtools/Venn/).

## Statistics

Power analysis was performed using G*Power software (version 3.1, Germany), alpha set as 0.05, which determined that 10 mice/group was required for statistical significance for behavioral testing.^24^ However, to account for animal deaths and exclusions, n = 15 was planned for behavioral testing, and all other experiments comprised n = 6–10. All data are presented as mean ± SD. Outliers were identified and removed with GraphPad Prism software (Version 10.0.3, GraphPad Software Inc., USA), using the robust regression and outlier removal method, coefficient Q = 1%. Groups were tested for normal or lognormal distribution using the Shapiro-Wilk or D’Agostino & Pearson test in GraphPad Prism 10, as appropriate. Statistical analyses performed using unpaired t-test, two-way ANOVA (mixed-effects) with Tukey’s multiple comparisons test, Kruskal-Wallis test, or an aligned ranked transformed (ART) ANOVA (mixed-effects) with Tukey’s multiple comparisons, as appropriate. Data was analyzed using GraphPad Prism 10 or RStudio (Version 4.3.2, R Foundation for Statistical Computing, Austria). All groups were randomized with the investigator blinded to all procedures and analyses.

## Results

### Infarct volume and blood pressure and cerebral blood flow were not altered in comorbid mice

Systolic blood pressure (SBP) was comparable across all groups at day 0 (Figure 1(b) and (c)). Angiotensin II increased SBP to ≥140 mmHg at day 7, which remained elevated until day 14 (Figure 1(b)). There was a significant main effect of hypertension (14 days; *F*(3, 39) = 34.40, *P < *0.05), and a significant interaction between hypertension and time (14 days; *F*(3,39) = 15.99, *P < *0.05). Angiotensin II increased SBP to ≥140 mmHg at day 7, which remained elevated until day 28 (Figure 1(c)). There was a significant main effect of hypertension (28 days; *F*(3,116) = 79.34, *P < *0.05), and a significant interaction between hypertension and time (28 days; *F*(6,116) = 28.06, *P < *0.05). Stroke induction at day 7 had no effect on SBP in any group (*P > *0.05). There was no significant differences in infarct volume between the 14 day NT (4.2 ± 3.6 mm^3^) and HT (6.0 ± 3.3 mm^3^) mice (Figure 1(d); *t*(16) = 1.10, *P > *0.05). There was also no significant differences in infarct volume between the 28 d NT (Figure 1(e); 2.3 ± 1.1 mm^3^) and HT (2.1 ± 1.3 mm^3^) (*t*(21) = 0.42, *P < *0.05). Stroke was the main effect for reduced cortical CBF at day 14 in both normotensive and hypertensive mice (*F*(1,27) = 5.92, *P < *0.05, 95% CI of difference [2.48, 29.17]), however no significant interaction was observed *(F*(1,27) = 0.87, *P = *0.36*;* Supplementary Figure 1A–B). Within the infarct area, the relative change in CBF (compared with the matched area at baseline) was similar between normotensive and hypertensive mice (−28.3 ± 15.7% vs. −27.9 ± 13.6%; *t*(13) = 0.06, *P > *0.05, 95% CI [−15.92, 16.81]; Supplementary Figure 1A and C).

### Spontaneous hemorrhages were more common in comorbid mice

Post-stroke hemorrhage was macroscopically observed in mice at 14 days but not 28 days. We observed hemorrhage in one mouse from the NT + Stroke group, which was distal to the infarct area, whereas hemorrhages were observed in 5 mice from the HT + Stroke group (4 distal and 1 local) (Figure 1(f); *P < *0.05). An illustrative example of the scoring shown in Figure 1(f), right.

### Spatial memory was impaired after hypertension but not stroke

#### Object relocation task

**Figure 2.**
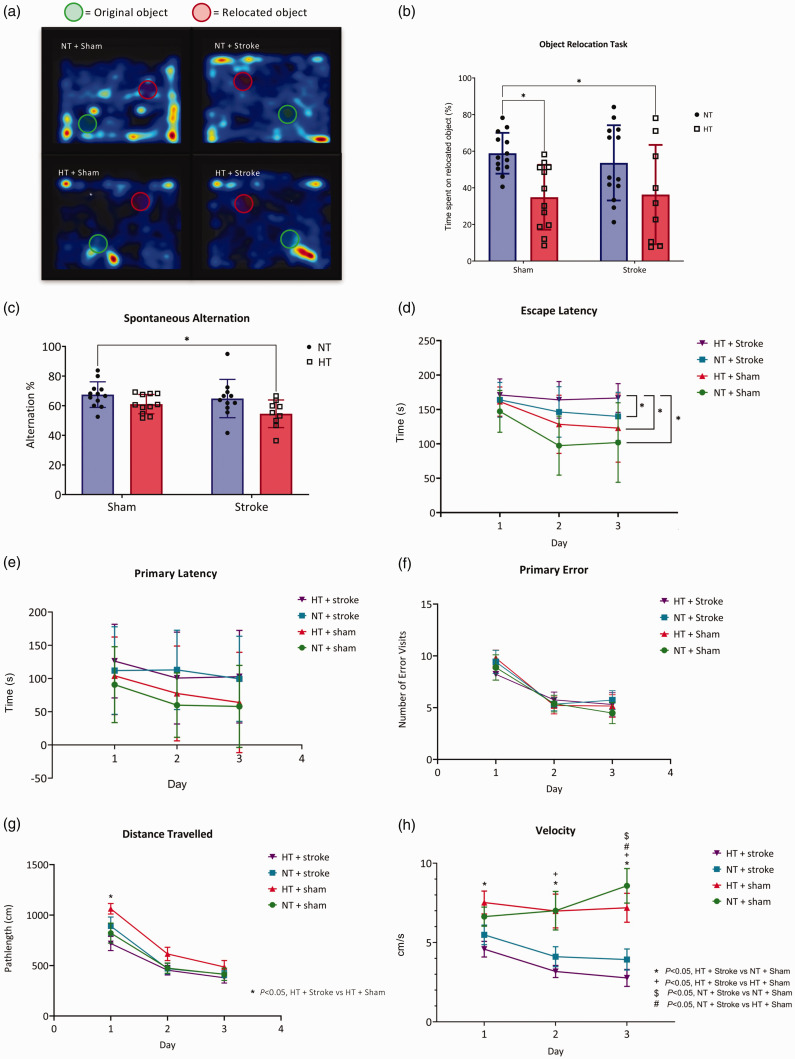
The assessment of memory and learning. (a) Representative heat maps of activity when performing the object relocation task with the location of the original and relocated object shown in mice undergoing the 14-day protocol. (b) Quantification of the % time spent interacting with the relocated object. (c) Spontaneous alternation test conducted using the Y maze in mice undergoing the 14-day protocol. The Barnes maze was performed in mice undergoing the 28-day protocol. (d) Escape latency, (e) primary latency (s), (f) primary error, (g) distance travelled (cm), and (h) velocity (cm/s) were measured across 3 days. (b) Data are presented as mean ± SD, n = 9–13/group, (c) and n = 9–12/group. (b and c) * = *P < *0.05, two-way ANOVA mixed-effects with Tukey’s multiple comparisons test. (d–h) Data are presented as mean ± SD, n = 12–13/group. (d and f) * = *P < *0.05, aligned ranked transformed ANOVA mixed-effects with Tukey’s multiple comparison test and (e, g and h) two-way ANOVA repeated-measures with Tukey’s multiple comparison test.

Spatial working memory was evaluated using the object relocation task (14-day timepoint). NT + Sham (control) mice spent 59 ± 11% of the time interacting with the relocated object. In comparison, HT + Sham mice spent 35 ± 18% and HT + Stroke spent 36 ± 27% with the relocated objects (Figure 2(a) and (b); 95% CI of difference [3, 45] and 95% CI of difference [0.3, 45], respectively). There was a statistically significant effect of hypertension on the % of time spent with the relocated object (*F*(1,43) = 13, *P < *0.05). There was no significant difference between the NT + Sham and NT + Stroke (54 ± 21%; Figure 2(a) and (b); *P > *0.05).

#### Spontaneous alternation test

Spatial working memory was assessed using the spontaneous alternation test (14-day timepoint). There was no significant effect of stroke (*P > *0.05). However, there was a statistically significant effect of hypertension on performance in the spontaneous alternation task (*F*(91,39) = 7.99, *P < *0.05). Compared to NT + Sham (67 ± 9%), only the HT + Stroke (55 ± 9%) group had a significantly reduced rate of spontaneous alternation, (Figure 2(c); 95% CI of difference [1.54, 24,32], *P < *0.05).

### The combination of hypertension and stroke worsens cognition

Learning and memory was evaluated via the Barnes maze in mice undergoing 28-day timepoint. Escape latency (time to enter the escape hole) was lower in NT + Sham, HT + Sham and NT + Stroke groups over the 3-day testing period (Figure 2(d)). There was a significant effect of hypertension on escape latency (*F*(1, 150) = 13.91, *P < *0.05). Furthermore, there was a significant effect of stroke on escape latency (*F*(1,150) = 29.39, *P < *0.05). The escape latency on day 3 for NT + Stroke (140 ± 35 s) mice was greater than NT + Sham (102 ± 59 s), (Figure 2(d); CI 95% of difference [−56.41, −11.50], *P < *0.05). By contrast, the HT + Stroke group took 167 ± 21 s to enter the escape hole, significantly longer than the NT + Stroke (95% CI of difference [−38.90, 6.00]), HT + Sham (123 ± 50 s; 95% CI of difference [−52.25, −7.35]), and NT + Sham mice (Figure 2(d); 95% CI of difference [−72.86, −27.95], *P < *0.05). Primary latency, and primary errors were comparable for all groups across three days (Figure 2(e) and (f); *P > *0.05). However, the distance travelled at day 1 was significantly shorter in the HT + Stroke group (718.3 ± 244.0 cm) when compared to HT + Sham (1062.1 ± 188.5 cm; CI of difference [−588.6, −98.99]). Otherwise, the distance travelled was not significantly different across all groups for the second and third day of testing (Figure 2(g); *P > *0.05). There was a significant stroke effect on velocity when compared with sham treated mice (Figure 2(h); *F*(3, 44) = 12.00, *P < *0.05). The velocity of HT + Stroke group (2.8 ± 1.9 cm/s) was significantly lower than HT + Sham (7.2 ± 3.3 cm/s; 95% CI of difference [−7.00, −1.00], *P < *0.05) and NT + Sham groups (7.2 ± 3.9 cm/s; 95% CI of difference [−9.00, −3.00], *P < *0.05) at day 3. Additionally, the velocity of NT + Stroke group (3.9 ± 2.3 cm/s) was significantly lower than HT + Sham (95% CI of difference [−6.00, 0.30], *P < *0.05) and NT + Sham groups (95% CI of difference [−8.00, −1.00], *P < *0.05) at day 3. However, there was no difference in velocity between HT + Stroke vs NT + Stroke across all days (*P > *0.05).

### Post-stroke blood-brain barrier permeability was not increased with hypertension

**Figure 3.**
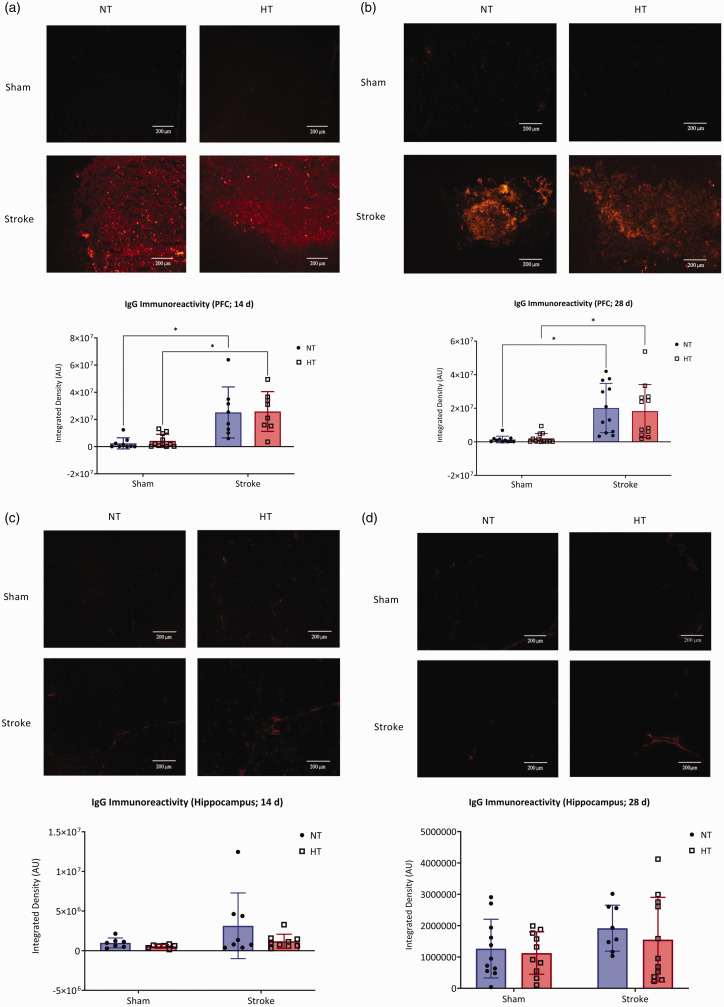
Blood-brain barrier integrity. Representative photomicrographs (top) of IgG in the prefrontal cortex across all groups for the (a) 14-day protocol and (b) 28-day protocol. Images were taken at 400X magnification, images were taken on the edge of the Continued.peri-infarct region and equivalent locations were then imaged in sham mice. Quantification of IgG integrated density (bottom) across all groups in the (a) 14-day protocol, and (b) 28-day protocol. Representative photomicrographs (top) of IgG in the hippocampus across all groups for the (c) 14-day protocol, (d) and the 28-day protocol. Images taken at 400X magnification. Images were taken at comparable locations in all groups. Quantification of IgG integrated density (bottom) across all groups in the (c) 14-day protocol, (d) and the 28-day protocol. (a and b) Data are presented as mean ± SD, n = 7–12/group. * indicates *P < *0.05, analysis by two-way ANOVA mixed-effects with Tukey’s multiple comparisons test (c and d) Data are presented as mean ± SD, n = 7–11/group. Analysis by two-way ANOVA mixed-effects with Tukey’s multiple comparisons test.

IgG deposition was elevated after stroke in the prefrontal cortex of mice at both the 14- and 28-day timepoints and this effect was independent of blood pressure status (Figure 3(a) and (b)). At 14 days, there was no effect of HT on IgG deposition (*P > *0.05). Rather, stroke significantly increased IgG deposition in the infarct region, (Figure 3(a) and (b); *F*(1,30) = 40.60; *P > *0.05). Similarly at 28 days, no effect of HT was seen on IgG deposition (*P > *0.05). However, a significant effect of stroke on IgG deposition was observed at the 28 day timepoint (*F*(1,42) = 32.15, *P < *0.05). Hippocampal IgG deposition was not different between groups at either 14 or 28 days (Figure 3(c) and (d); *P > *0.05).

### Post-stroke reactive astrogliosis was not increased with hypertension

**Figure 4.**
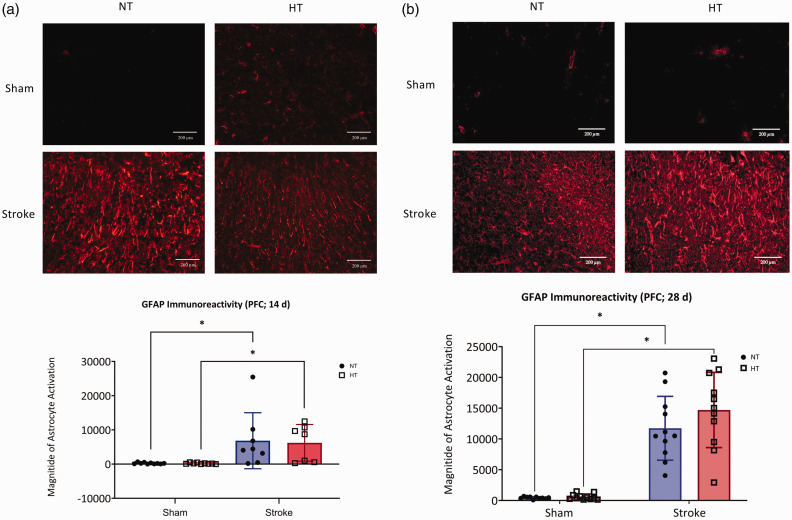
Astrocyte reactivity. Representative photomicrographs (top) of GFAP immunoreactivity in the prefrontal cortex at (a) 14-day and (b) 28-day. Images were taken at 400X magnification, images were taken on the edge of the peri-infarct region and equivalent locations were then imaged in shams. Quantification of astrocyte activation across all groups (bottom) in the (a) 14-day and (b) 28-day protocol. Data are presented as mean ± SD, (a) n = 7–10/group and (b) n = 10–11/group, * indicates *P < *0.05, analysis by (a) aligned ranked transformed ANOVA with Tukey’s multiple comparisons test and (b) two-way ANOVA with Tukey’s multiple comparisons test.

At 14 days, stroke increased astrocyte reactivity in the prefrontal cortex (Figure 4(a); *F*(1,30) = 15.51, *P < *0.05). There was no effect of hypertension on astrocyte reactivity (Figure 4(a); *P > *0.05). Similarly at 28 days, stroke was the main effect for astrocyte reactivity (Figure 4(b); *F*(1,36) = 37.27, *P < *0.05). No effect of hypertension on astrocyte reactivity was observed, (Figure 4(b): *P > *0.05).

### Stroke increases astrocytic and microglial inflammatory markers within the brain

Stroke increased gene expression of the proinflammatory A1 astrocyte markers *Serping1*, *Psmb8*, *Srgn*, and *Gbp2* in both normotensive and hypertensive mice (Supplementary Figure 2A; *P < *0.05). The A1 astrocyte marker *Srgn* was increased in HT + Stroke vs HT + Sham (Supplementary Figure 2A; *P < *0.05). Stroke was also the main effect in increasing the anti-inflammatory A2 astrocyte markers: *Cd14* and *Clcf1*, whereas *Tgm1* was only increased in HT + Stroke vs HT + Sham (Supplementary Figure 2B; *P < *0.05). Gene expression of the proinflammatory M1 microglia marker *Stat3* was significantly increased in NT stroke vs NT Sham (Supplementary Figure 2C; *P < *0.05), whereas expression of *Tnfa* and *Nos2* were not changed (Supplementary Figure 2C). M2 microglia markers *Il4*, *Arg1*, and *Fam19a3* (Supplementary Figure 2D) were not changed by either hypertension or stroke.

### Neuroinflammatory gene expression was increased in comorbid mice

**Figure 5.**
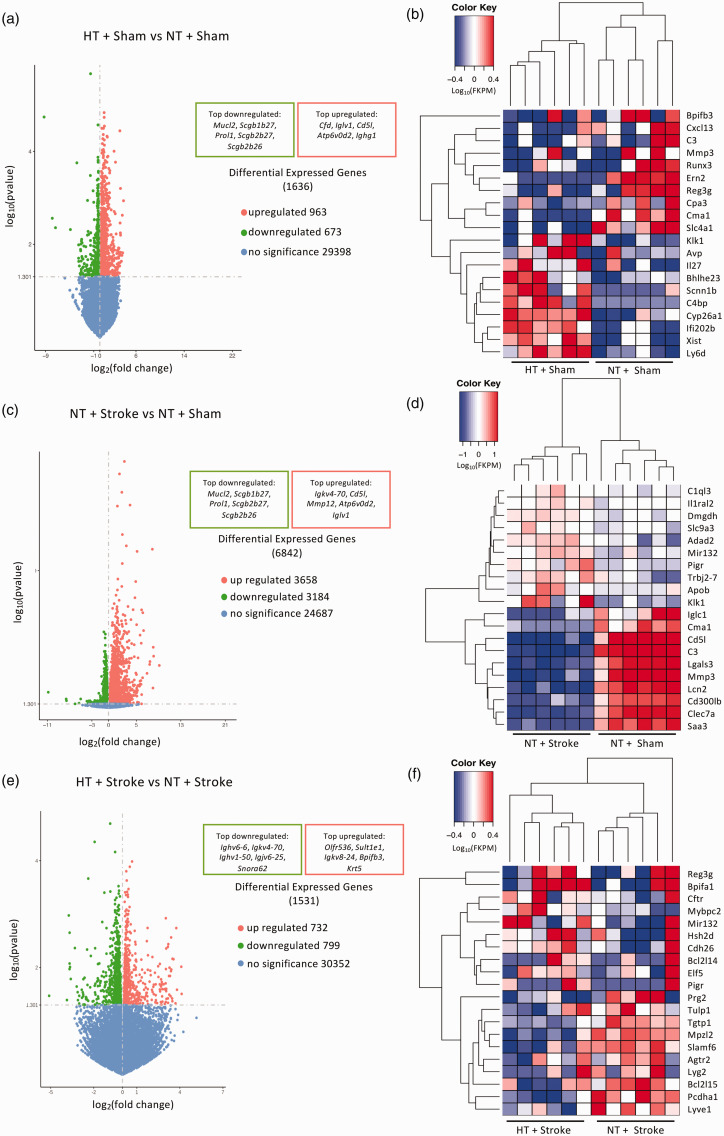
Transcriptomic analysis of hypertensive and stroke brains within the prefrontal cortex. Volcano plots of the log_10_ adjusted p-value vs log_2_ fold change of differentially expressed genes in the (a) hypertensive (HT) + Sham vs normotensive (NT) + Sham, (c) NT + Stroke vs NT + Sham, and (e) HT + Stroke vs normotensive stroke NT + Stroke comparison. Heat map analysis of the top 10 up- and downregulated neuroinflammatory genes in the (b) HT + Sham vs NT + Sham, (d) NT + Stroke vs NT + Sham, and (f) HT + Stroke vs NT + Stroke comparison. Gene expression is shown as log_10_(FPKM +1) with a p-value threshold of <0.05.

The distribution of 1636 DEGs (963 upregulated and 673 downregulated) in the brains of HT + Sham vs NT + Sham is shown in Figure 5(a). The top 10 upregulated genes include the proinflammatory gene interleukin 27 (*Il27*), and top downregulated genes included matrix metalloprotease 3 (*Mmp3*) which is important for blood-brain barrier integrity and permeability (Figure 5(b)).

The distribution of 6842 DEGs (3658 upregulated and 3184 downregulated) in the brains of NT +Stroke vs NT + Sham is shown in Figure 5(c), and the top 10 up- and downregulated genes are summarized in Figure 5(d). This included upregulation of apolipoprotein B (*Apob*) associated with atherosclerosis and ischemic stroke, and downregulation of complement 3 (*C3*) associated with the activation of the complement system which is vital for a diverse array of functions such as angiogenesis, neuronal maturation, and tissue regeneration.

The distribution of 1531 DEGs (732 upregulated and 799 downregulated) in the brains of HT + Stroke vs NT + Stroke mice is shown in Figure 5(e). The top 10 highly upregulated genes involved pro-apoptotic processes such as Bcl2-like 14 (*Bcl2l14*), hematopoietic SH2 domain containing protein (*Hsh2d*), neurodegeneration such as microRNA 132 (*Mir132*), and proinflammatory processes such as polymeric immunoglobulin receptor (*Pigr*) (Figure 5(f)). The top 10 downregulated genes included inflammasome-related and pro-apoptotic pathways such as Bcl2-like 15 (*Bcl2l15*), GTP activity such as T-cell specific GTPase 1 (*Tgtp1*), and anti-inflammatory pathways, i.e. angiotensin II type 2 receptor (*Agtr2*) (Figure 5(f)).

### Expression of genes related to extracellular matrix, immunity and cognition

Gene ontology enrichment analysis found the top 20 significantly up- and downregulated GO terms related to various biological processes for the comparisons between hypertensive vs normotensive mice, stroke vs sham, and hypertensive stroke vs normotensive sham.

**Figure 6.**
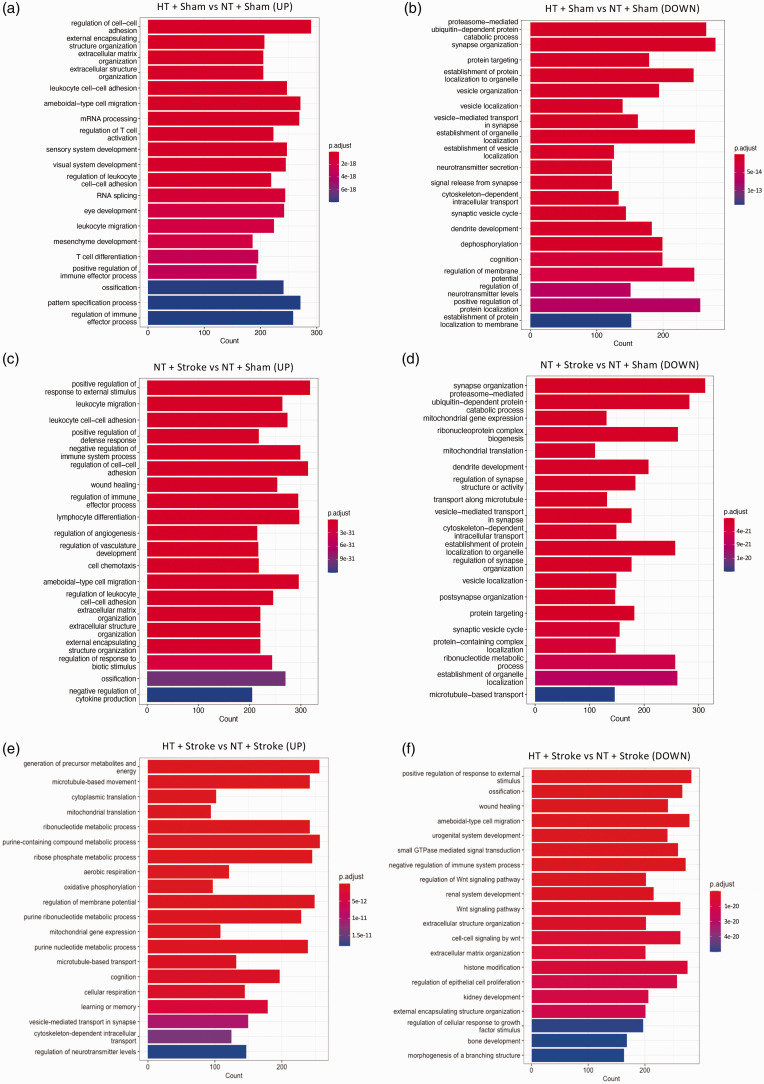
Gene ontology enrichment analysis of hypertensive and stroke brains. The GO terminology enriched in the set of genes that encode for the up- and downregulated processes related to biological processes of the (a and b) hypertensive (HT) + Sham vs normotensive (NT) + Sham, (c and d) NT + Stroke vs NT + Sham, and (e and f) HT + Stroke vs NT + Stroke within the prefrontal cortex. GO terms are ordered by their p-adjusted values, threshold was set to <0.01. Length of GO bars indicate the number of genes related to their corresponding GO term, color key represents the degree of significance (p-adjusted value).

The upregulated GO enrichment analysis comparing HT + Sham vs NT + Sham revealed 205 genes related to *extracellular matrix organization*, and 223 genes related to the *regulation of T cell activation* (Figure 6(a)). The top DEGs associated with *extracellular matrix organization* were: *Cma1*, *Mmp12*, and *Mmp3*. Furthermore, the top DEGs associated with the *regulation of T cell activation* were: *Slca4a1*, *Runx3*, and *Lgals3*. The downregulated GO enrichment analysis found 279 genes related to *synapse organization*, 183 genes related to *dendrite development*, and 199 genes related to *cognition* (Figure 6(b)). The top downregulated genes associated with *synapse organization* included: *Adgrf1*, *Disc1*, and *Ghsr*. The top downregulated genes associated with *dendrite development* included: *Cacna1f*, *Disc1*, and *Trpc6*. Lastly, the top downregulated DEGs associated with *cognition* included: *Ttc36*, *Adam2*, and *Grpr*.

Similarly, analysis of upregulated GO terminology comparing NT + Stroke vs NT + Sham revealed 299 genes related to the *negative regulation of immune system process*, and 221 genes related to *extracellular matrix organization* (Figure 6(c)). The top DEGs associated with the *negative regulation of immune system process* included: *Mmp12*, *Lgals3*, and *Cst7*. Moreover, the top DEGs associated with the *extracellular matrix organization* included: *Mmp12*, *Mmp3*, *Lgals3*. As for the downregulated GO terminologies, 312 genes were related to *synapse organization*, and 208 genes were related to *dendrite development* (Figure 6(d)). The top downregulated genes associated with *synapse organization* included: *Adgrf1*, *C1ql3*, and *Il1rapl2*. Lastly, the top downregulated genes associated with *dendrite development* included: *Mir132*, *Atp7a*, and *Fezf2*.

**Figure 7.**
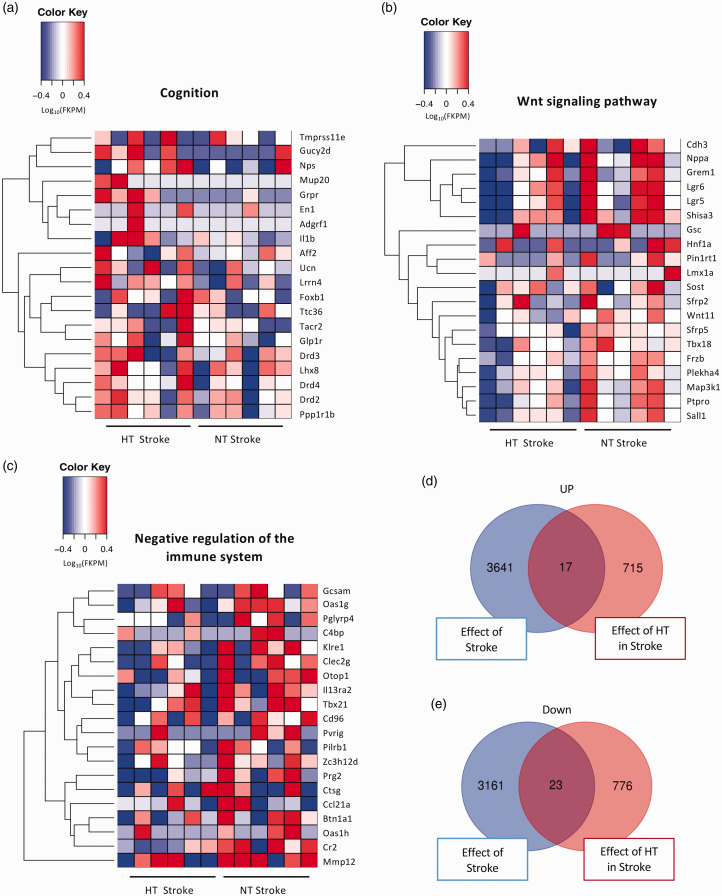
Heatmap analysis of the hypertensive stroke brain. The top 20 differentially expressed genes from the hypertensive (HT) + Stroke vs normotensive (NT) + Stroke comparison determined by gene ontology enrichment analysis: (a) cognition, (b) Wnt signaling pathway and (c) negative regulation of the immune system. Gene expression is shown as log_10_(FPKM +1) with a p-value threshold of <0.05. Venn diagrams illustrate the number of: (d) upregulated and (e) downregulated differentially expressed genes that are unique and shared between the NT + Stroke vs NT + Sham (effect of stroke) and HT + Stroke vs NT + Stroke (effect of hypertension in stroke) comparisons.

GO terminology comparing HT + Stroke vs NT + Stroke groups found 197 upregulated genes related to *cognition* (Figure 6(e)). The top DEGs associated with *cognition* included: *Grpr*, *Mup20*, and *Gucy2d*. Moreover, there were more than 202 downregulated genes related to *Wnt signaling* and 272 downregulated genes related to *negative regulation of immune processes* (Figure 6(f)). The top downregulated genes associated with *Wnt signaling* included *Pin1rt1*, *Nppa*, and *Shisa3*. The top downregulated genes associated with *negative regulation of immune processes* were *Prg2*, *Tbx21*, and *Pvrig*. Heat map analysis of the top 20 DEGs based on the GO enrichment analysis comparing HT + Stroke vs NT + Stroke related to *cognition*, *Wnt signaling pathway*, and *negative regulation of the immune system* is shown in Figure 7. The number of upregulated and downregulated DEGs that are unique or common to the comparison between NT + Stroke vs NT + Sham (effect of stroke) and HT + Stroke vs NT + Stroke (effect of hypertension in stroke) is shown in Figure 7(d) and (e), respectively.

## Discussion

The findings of this study support the hypothesis that hypertension exacerbates post-stroke cognitive impairment. There are several important findings of the study. First, we identified that hypertension and stroke alone impaired aspects of cognitive function in mice. Second, the combination of hypertension and stroke exacerbated the impairment of executive function and increased the occurrence of spontaneous brain hemorrhage. Third, hypertension or stroke alone altered the genetic landscape in brain and comorbidity resulted in further genetic changes which may contribute to the worsening of outcomes. Our findings highlight the profound impact that hypertension and stroke, alone or in combination, have on the brain.

We found impaired spatial working memory in hypertensive mice, but not stroke alone, using the object relocation task.^20^ Moreover, using the spontaneous alternation test to assess spatial working memory, there was no effect of stroke or hypertension alone one-week post-stroke. These findings are consistent with previous studies, in which no memory impairment was observed one week after prefrontal stroke.^19^ Importantly, impaired working memory was only observed with the combination of hypertension and stroke when compared to control. This finding is consistent with worse outcomes in comorbid mice. Next, we assessed spatial reference memory and learning in mice at an extended time-point using the Barnes maze. Distance travelled and primary error were all comparable across all groups which indicates interaction with the behavioral task. There was also no significant effect on primary latency (time taken to locate the escape hole), indicating mice are able to locate and navigate to the escape hole in a similar time. However, we observed a profound impairment in the latency to enter the escape hole. While no apparent impairment was observed in hypertension alone, stroke alone resulted in impaired cognition across the 3 days. More importantly, the combination of stroke and hypertension exacerbated the impairments as shown by a longer escape latency when compared to other groups. Overall, our findings suggest that executive function is impaired by stroke in the prefrontal cortex and the combination of hypertension and stroke exacerbates this impairment. Indeed, it has previously been shown that when mice enter the escape hole, there is an increased firing of prefrontal cortex neurons whereas this not observed when animal located the escape hole,^25^ highlighting the critical role of the prefrontal cortex in this process. While previous studies have reported spatial memory impairment with the Barnes maze at 30 d after the induction of hypertension,^16^ that study used a higher dose of angiotensin II (0.86 vs 0.28 mg/kg/d). As angiotensin II has several direct effects on the cerebral vasculature independent of hypertension,^14^ a higher dose may have promoted a greater impairment of spatial memory. Despite worsened cognition, we found no difference in infarct volume between normotensive and hypertensive mice following stroke at either the 14- or 28-day timepoints. While studies in other models of hypertension and stroke have reported larger infarcts (e.g. larger infarcts in stroke-prone spontaneously hypertensive rats compared Wistar-Kyoto rats^26^) our findings suggest that hypertension does not contribute to the size of the infarct in this model of stroke. Thus, mechanisms not related to direct brain injury contribute to the worsened cognition in comorbid mice.

A previous study has reported that angiotensin II levels are elevated in both the ipsilateral and contralateral hemispheres after MCAO.^27^ While it is unknown whether a similar increase occurs after PT stroke, we note that in the present study, systolic blood pressure was not affected by stroke. Thus, any change to angiotensin II levels after stroke, if present, was not sufficient to change blood pressure. This is perhaps not surprising considering the reported levels of angiotensin II in the brain after stroke (242 pg/g of tissue) is considerably lower than the dose we delivered via osmotic minipump.^27^ Secondly, we targeted the stroke to the prefrontal cortex which is relatively distant to key blood pressure controlling regions of the brain that are activated by angiotensin II during hypertension (e.g. subfornical organ, circumventricular organs, hypothalamus, and brainstem).^28^ However, we can’t exclude the possibility that the increased disease severity in comorbid mice (increased haemorrhages and cognitive impairment) may be due to blood pressure independent effects of angiotensin II. This possibility warrants future examination.

We observed extravasation of IgG – the most abundant antibody class in plasma – in the prefrontal cortex of all mice subjected to stroke, indicating a compromised BBB following cerebral ischemia. However, IgG extravasation was no more severe in post-stroke mice that were also hypertensive. Nevertheless, hypertension markedly increased the incidence of spontaneous macroscopic brain hemorrhages in mice that had received stroke. Clinically, hemorrhagic transformation occurs in up to 40% of ischemic stroke cases.^29^ The occurrence of hemorrhages both within and distal to the infarct region may be related to events following the stroke, such as global inflammation and oxidative stress, which can contribute to the development of endothelial dysfunction and subsequent vessel rupture.^30^ Interestingly, we only observed hemorrhagic transformation in mice in the 14-day protocol, despite similar elevations in systolic blood pressure as in mice belonging to the 28-day protocol. Although, the underlying mechanisms are unclear, the multitude of effects that angiotensin II exerts on the cerebral circulation, including disruption of BBB proteins,^14^ may have possibly contributed to this effect.

The polarization of astrocytes and microglia were of interest as glial cells play a major role in modulating neuroinflammation,^31^ which we hypothesize is a contributing factor to worsened outcomes after hypertension and stroke. We observed an overall elevation of proinflammatory and anti-inflammatory astrocyte and microglia markers in stroke mice at day 28. However, the cause of exacerbated cognitive impairment in comorbid mice remains to be elucidated. Thus, we analyzed transcriptomic changes in the brains of mice subjected to stroke and/or hypertension with a focus on neuroinflammatory genes. The worsened cognitive function observed with the combination of hypertension and stroke was associated with an increase expression of genes which contribute to apoptotic and inflammatory pathways. For example, we found changes in expression of genes belonging to Bcl-2 family proteins associated with the inflammasome complex to induce apoptosis including upregulation of *Bcl2l14*. By contrast, *Bcl2l15*, another pro-apoptotic gene, was downregulated. Opposing expression patterns in apoptotic markers could be explained by *Bcl2l14* assuming another role such as intracellular protein trafficking rather than stress-induced apoptosis.^32^ This is consistent with the concept that apoptosis predominantly occurs in acute stages (<48 h) of stroke^33^ and that repair and plasticity occur in chronic stages.^34^

There is increasing relevance of microRNAs as biomarkers for stroke pathology.^35^ Indeed, it has been suggested that miR-132 may be a biomarker for mild and post-stroke cognitive impairment,^36,37^ and overexpression of miR-132 impairs neuroplasticity and short term memory.^38^ We found *Mir132* gene expression was upregulated after stroke in normotensive mice compared with sham and was further upregulated in hypertensive mice after stroke compared with normotensive mice after stroke. Our findings are consistent with the aforementioned studies showing increased miR-132 is associated with worsened outcomes. However, the role of miR-132 is complex with other studies showing that miR-132 reduces β-amyloid accumulation and drives neurogenesis in the hippocampus.^39,40^ Thus, it is possible that the upregulation of *Mir132* gene expression in the brains of hypertensive mice after stroke may be a compensatory response in order to promote repair of the injured tissue. Further studies are needed to clarify the role of miR-132 in the setting of hypertension and stroke.

Our RNA sequencing analysis revealed that gene expression of the angiotensin II type 2 receptor (AT2R), *Agtr2*, was downregulated in HT + Stroke compared to NT + Stroke. AT2R has been suggested to play a role in cognition,^41^ and induces anti-inflammatory effects by promoting anti-inflammatory cytokine, such as IL-10, release.^42^ Furthermore, activation of the AT2R promotes neurite outgrowth and supports neuronal survival in the brain during ischemia.^43^ Thus, downregulation of the AT2R after hypertension and stroke may result in loss of anti-inflammatory and neuronal survival pathways, leading to worsened outcomes. GO enrichment analysis revealed an upregulation of genes that negatively influence cognition, such as dopamine receptor D4 (*Drd4*) which inhibits adenylyl cyclase and has been implicated in neurobehavioural disorders,^44^ and interleukin 1 beta (*Il1b*) which encodes for the proinflammatory cytokine IL-β which is produced by caspase-1 and is involved in pyroptosis with downstream effects that likely have a negative impact on cognition.

GO enrichment analysis revealed Wnt signaling pathways to be downregulated in HT + Stroke group when compared to NT + Stroke. Wnt signaling plays a vital role in many processes in cells of the CNS including survival, proliferation and function of the cerebrovascular endothelial cells, neurons, pericytes, astrocytes, microglia, and oligodendrocytes.^45^ Canonical Wnt/β-catenin signaling has been reported in stroke and may act as a neuroprotective and regenerative factor,^46^ and in the promotion hippocampal neurogenesis.^47^ Our finding of downregulation of Wnt signaling in HT + Stroke mice could lead to attenuation of neuroprotective signaling mechanisms and thereby limit post-stroke repair.

Overall, our data indicate that hypertension and stroke may each result in cognitive impairment which is exacerbated when these diseases occur in combination. Thus, our findings highlight the critical importance of treating hypertension effectively, especially in stroke patients, to limit cognitive decline and dementia risk.

## Supplemental Material

sj-pdf-1-jcb-10.1177_0271678X241262127 - Supplemental material for Post-stroke cognitive impairment and brain hemorrhage are augmented in hypertensive mice Supplemental material, sj-pdf-1-jcb-10.1177_0271678X241262127 for Post-stroke cognitive impairment and brain hemorrhage are augmented in hypertensive mice by David E Wong Zhang, Tayla A Gibson Hughes, Hericka B Figueiredo Galvao, Cecilia Lo, Quynh Nhu Dinh, Shenpeng R Zhang, Hyun Ah Kim, Sharmalee Selvaraji, Andrew N Clarkson, Thiruma V Arumugam, Grant Drummond, Christopher G Sobey and T Michael De Silva in Journal of Cerebral Blood Flow & Metabolism

## Data Availability

The data that support the findings of this study are available from the corresponding author upon reasonable request. The datasets for bulk RNA sequencing discussed in this publication have been deposited in NCBI’s Gene Expression Omnibus^48^ and are accessible through GEO Series accession number GSE247789 (https://www.ncbi.nlm.nih.gov/geo/query/acc.cgi?acc=GSE247789)
