# Supplementary material for: Post-stroke cognitive impairment and brain hemorrhage are augmented in hypertensive mice
Source: J Cereb Blood Flow Metab. 2024 Jun 17;44(12):1517–34. doi: 10.1177/0271678X241262127 (PMC11572097; doi:10.1177/0271678X241262127)
Supplement: sj-pdf-1-jcb-10.1177_0271678X241262127 - Supplemental material for Post-stroke cognitive impairment and brain hemorrhage are augmented in hypertensive mice [file sj-pdf-1-jcb-10.1177_0271678X241262127.pdf]

SUPPLEMENTARY INFORMATION

Post-Stroke Cognitive Impairment and Brain Hemorrhage are Augmented in Hypertensive Mice

David E. Wong Zhang[1,2], Tayla A. Gibson Hughes[1,2], Hericka B. Figueiredo Galvao[1,2], Cecilia Lo[2], Quynh Nhu Dinh[1,2], Shenpeng R. Zhang[1,2], Hyun Ah Kim[1,2], Sharmalee Selvaraji[3-5], Andrew N. Clarkson[2], Thiruma V. Arumugam[1,2], Grant Drummond[1,2], Christopher G. Sobey[1,2] and T. Michael De Silva[1,2]

[1]Centre for Cardiovascular Biology and Disease Research and La Trobe Institute for Molecular Sciences (LIMS), La Trobe University, Victoria, Australia;

[2]Department of Microbiology, Anatomy, Physiology & Pharmacology, School of Agriculture, Biomedicine, Environment, La Trobe University, Victoria, Australia;

[3]Department of Physiology, Yong Loo Lin School Medicine, National University of Singapore, Singapore, Singapore

[4]Memory Aging and Cognition Centre, Department of Pharmacology, Yong Loo Lin School of Medicine, National University of Singapore, Singapore, Singapore

[5]NUS Graduate School for Integrative Sciences and Engineering, National University of Singapore, Singapore, Singapore

Corresponding author: T. Michael De Silva, PhD

Centre for Cardiovascular Biology and Disease Research and

La Trobe Institute for Molecular Sciences (LIMS)

Department of Microbiology, Anatomy, Physiology & Pharmacology

School of Agriculture, Biomedicine, Environment

La Trobe University

Bundoora, Victoria 3086, Australia

e-Mail: t.desilva@latrobe.edu.au

Phone: +61-3-94796876

Table 1. Experimental groups

Groups	0.7 mg/kg/d; 14 d protocol	0.28 mg/kg/d; 28 d protocol
NT + Sham	N=10	N=12
NT + Stroke	N=10	N=9
HT + Sham	N=11	N=12
HT + Stroke	N=12	N=13

Supplementary Table 1. Summary of experimental groups. Hypertension (HT; angiotensin II 0.7mg/kg/d or 0.28 mg/kg/d) or normotension (NT) group in combination with either sham or stroke surgery.

Table 2. Primers used for Real-Time PCR

Gene	Assay ID
Arg1	Mm00475988_m1
Cd14	Mm00438094_g1
Clcf1	Mm01236492_m1
EMP1	Mm00515678_m1
FAM19A3	Mm01305353_m1
Gbp2	Mm00494575_m1
Il4	Mm00445259_m1
iNOS	Mm00440502_m1
Psmb8	Mm01278979_m1
Serping1	Mm00437835_m1
Srgn	Mm01169070_m1
Stat3	Mm01219775_m1
Tgm1	Mm00498375_m1

Supplementary Table 2. Primers used for real time PCR. Assay ID's for genes measured by real time PCR.

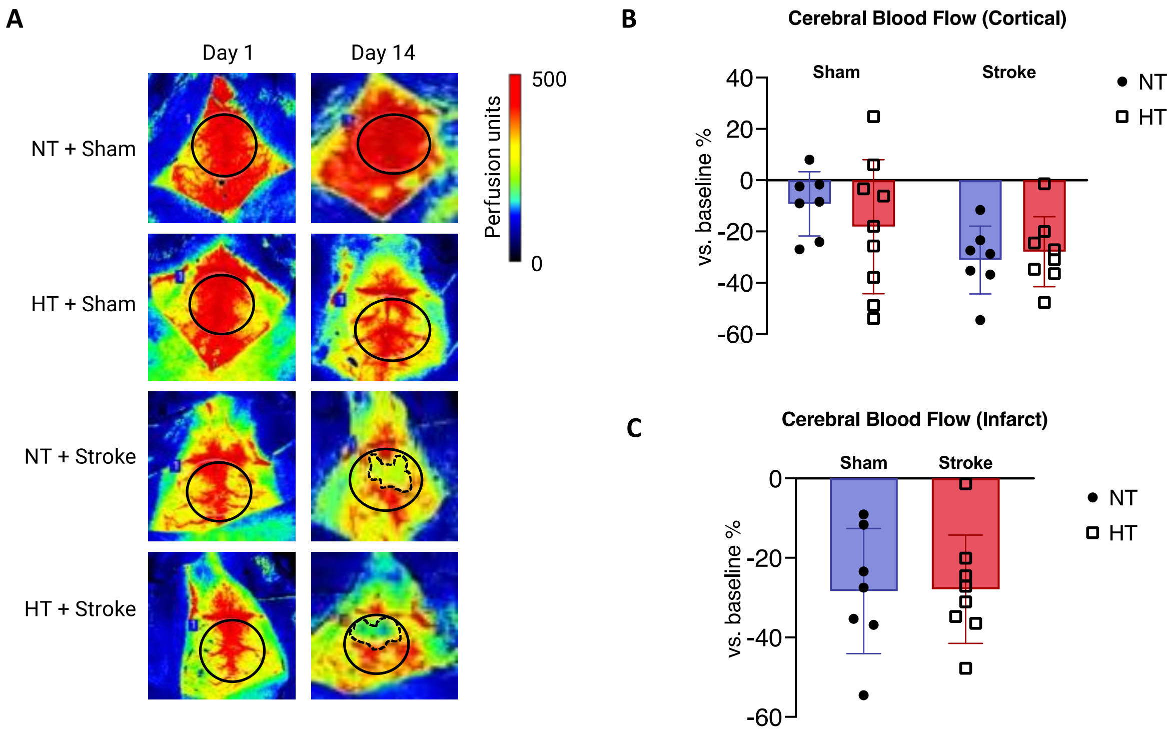

Supplementary Figure 1. Cerebral blood flow in mice from the 14-day protocol. (A) Representative lase speckle contrast images of cerebral blood flow (CBF) of each group at baseline (day 0) and prior to euthanasia (day 14). Cortical CBF was measured in a 20 mm^2 area shown by the solid line and summarised in **(B)**. The area of perfusion deficit (infarct area) is shown by the dashed line and summarised in **(C)**. **C**erebral blood flow at day 14 is compared to the same area measured at day 0. Data is presented as mean ± SD, n=8-9/group. Two-way ANOVA.

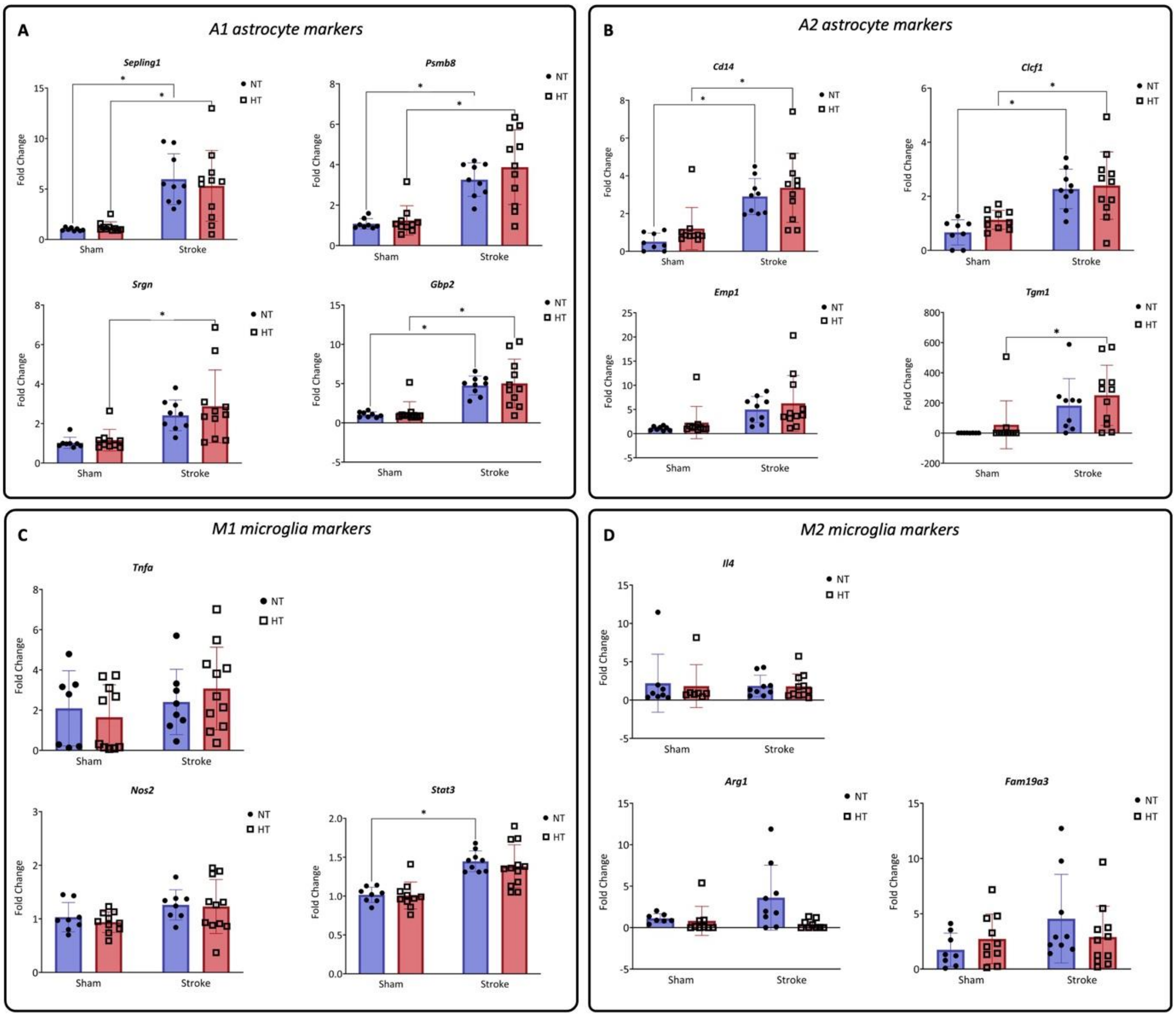

Supplementary Figure 2. Expression of astrocytic and microglial markers in the brain. Classical markers of the **(A)** proinflammatory A1 astrocytes and **(B)** anti-inflammatory A2 astrocytes; **(C)** proinflammatory M1 microglia and **(D)** anti-inflammatory M2 microglia. Data is presented as mean ± SD, n=6/group. * Indicates *P*<0.05, two-way ANOVA with Tukey's multiple comparisons test.
